# Supplementary material for: HIVprotI: an integrated web based platform for prediction and design of HIV proteins inhibitors
Source: J Cheminform. 2018 Mar 9;10:12. doi: 10.1186/s13321-018-0266-y (PMC5845081; doi:10.1186/s13321-018-0266-y)
Supplement: Supplementary file 2 — Additional file 2. Source code of HIVProtI web server. [file 13321_2018_266_MOESM2_ESM.zip › HIVprotI_Source-code/hivprotI/weka-3-6-6/doc/weka/core/RevisionHandler.html]

RevisionHandler


---


|  |  |  |
| --- | --- | --- |
| **Overview** \| **Package** \| **Class** \| **Tree** \| **Deprecated** \| **Index** \| **Help** \| **Weka's home** | |  |
| **PREV CLASS**   **NEXT CLASS** | **FRAMES**    **NO FRAMES**     **All Classes** |  |
| SUMMARY: NESTED \| FIELD \| CONSTR \| METHOD | DETAIL: FIELD \| CONSTR \| METHOD |  |


---


## weka.core Interface RevisionHandler

**All Known Subinterfaces:**: ConditionalEstimator, Loader, Saver, Stemmer

**All Known Implementing Classes:**: AbstractAssociator, AbstractClusterer, AbstractDensityBasedClusterer, AbstractFileLoader, AbstractFileSaver, AbstractLoader, AbstractSaver, AbstractStringDistanceFunction, AbstractTimeSeries, AdaBoostM1, Add, AddClassification, AddCluster, AddExpression, AddID, AdditiveRegression, AddNoise, AddValues, ADNode, ADTree, Agrawal, AlgVector, AllFilter, AllJavadoc, AlphabeticTokenizer, AODE, AODEsr, Apriori, AprioriItemSet, ArffLoader, ArffLoader.ArffReader, ArffSaver, ASEvaluation, ASSearch, AssociatorEvaluation, Attribute, AttributeExpression, AttributeLocator, AttributeSelectedClassifier, AttributeSelection, AttributeSelection, AttributeSetEvaluator, AttributeStats, AttributeValueLiteral, AveragingResultProducer, Bagging, BallNode, BallSplitter, BallTree, BallTreeConstructor, BayesianLogisticRegression, BayesNet, BayesNet, BayesNetEstimator, BayesNetGenerator, BestFirst, BestFirst.Link2, BestFirst.LinkedList2, BFTree, BIFReader, BinarySparseInstance, BinC45ModelSelection, BinC45Split, BIRCHCluster, BMAEstimator, Body, BottomUpConstructor, BVDecompose, BVDecomposeSegCVSub, C45Loader, C45ModelSelection, C45PruneableClassifierTree, C45PruneableClassifierTreeG, C45PruneableDecList, C45Saver, C45Split, CachedKernel, Capabilities, CaRuleGeneration, Center, CfsSubsetEval, ChangeDateFormat, CharacterDelimitedTokenizer, ChebyshevDistance, Check, CheckAssociator, CheckAttributeSelection, CheckClassifier, CheckClusterer, CheckEstimator, CheckEstimator.AttrTypes, CheckEstimator.EstTypes, CheckEstimator.PostProcessor, CheckGOE, CheckKernel, CheckOptionHandler, CheckScheme, CheckScheme.PostProcessor, CheckSource, CheckSource, ChisqMixture, ChiSquaredAttributeEval, CholeskyDecomposition, CISearchAlgorithm, CitationKNN, ClassAssigner, ClassBalancedND, ClassDiscovery, ClassDiscovery.StringCompare, ClassificationGenerator, ClassificationViaClustering, ClassificationViaRegression, Classifier, ClassifierDecList, ClassifierSplitEvaluator, ClassifierSplitModel, ClassifierSubsetEval, ClassifierTree, ClassloaderUtil, ClassOrder, CLOPE, ClusterDefinition, ClusterEvaluation, ClusterGenerator, ClusterMembership, Cobweb, ComplementNaiveBayes, ConfusionMatrix, ConjunctiveRule, ConsistencySubsetEval, ConsistencySubsetEval.hashKey, ConsoleLogger, ContingencyTables, ConverterUtils, ConverterUtils.DataSink, ConverterUtils.DataSource, Copy, CorrelationSplitInfo, CostCurve, CostMatrix, CostSensitiveASEvaluation, CostSensitiveAttributeEval, CostSensitiveClassifier, CostSensitiveClassifierSplitEvaluator, CostSensitiveSubsetEval, CoverTree, CoverTree.CoverTreeNode, CrossValidationResultProducer, CSVLoader, CSVResultListener, CSVSaver, CVParameterSelection, Dagging, DatabaseConnection, DatabaseLoader, DatabaseResultListener, DatabaseResultProducer, DatabaseSaver, DatabaseUtils, DataGenerator, DataNearBalancedND, DBScan, DbUtils, DDConditionalEstimator, Debug, Debug.Clock, Debug.DBO, Debug.Log, Debug.Random, Debug.SimpleLog, Debug.Timestamp, DecisionStump, DecisionTable, DecisionTableHashKey, Decorate, DensityBasedClustererSplitEvaluator, DiscreteEstimator, DiscreteEstimatorBayes, DiscreteEstimatorFullBayes, DiscreteFunction, Discretize, Discretize, Distribution, DKConditionalEstimator, DMNBtext, DNConditionalEstimator, DoubleVector, DTNB, EditableBayesNet, EditDistance, EigenvalueDecomposition, Element, EM, END, EntropyBasedSplitCrit, EntropySplitCrit, Environment, EpsilonRange\_ListElement, Estimator, EstimatorUtils, EuclideanDistance, EuclidianDataObject, Evaluation, EvaluationUtils, ExhaustiveSearch, Experiment, ExponentialFormat, Expression, FarthestFirst, FastVector, FastVector.FastVectorEnumeration, FileLogger, Filter, FilteredAssociator, FilteredAttributeEval, FilteredClassifier, FilteredClusterer, FilteredSubsetEval, FindWithCapabilities, FirstOrder, FlexibleDecimalFormat, FloatingPointFormat, FlowRunner, FPGrowth, FromFile, FT, FTInnerNode, FTLeavesNode, FTNode, FTtree, GainRatioAttributeEval, GainRatioSplitCrit, GaussianPriorImpl, GaussianProcesses, GeneralizedSequentialPatterns, GeneralRegression, GeneticSearch, GeneticSearch, GeneticSearch, GlobalInfoJavadoc, GlobalScoreSearchAlgorithm, Grading, GraftSplit, GraphPanel, GreedyStepwise, GridSearch, Head, HierarchicalClusterer, HillClimber, HillClimber, HNB, HoldOutSubsetEvaluator, HyperPipes, IB1, IBk, ICSSearchAlgorithm, Id3, Impurity, IndividualInstance, IndividualInstances, IndividualLiteral, InfoGainAttributeEval, InfoGainSplitCrit, Instance, InstanceComparator, InstanceQuery, Instances, InstancesResultListener, InterquartileRange, IntVector, IsotonicRegression, ItemSet, IteratedLovinsStemmer, IteratedSingleClassifierEnhancer, J48, J48graft, Javadoc, JRip, Jython, K2, K2, KDConditionalEstimator, KDTree, KDTreeNode, KDTreeNodeSplitter, Kernel, KernelEstimator, KernelEvaluation, KernelFilter, KKConditionalEstimator, KMeansInpiredMethod, KOML, KStar, KStarCache, KStarCache.CacheTable, KStarCache.TableEntry, KStarNominalAttribute, KStarNumericAttribute, KStarWrapper, LabeledItemSet, LADTree, LAGDHillClimber, LaplacePriorImpl, LatentSemanticAnalysis, LBR, LBR.Indexes, LearningRateResultProducer, LeastMedSq, LED24, LFSMethods, LFSMethods.Link2, LFSMethods.LinkedList2, LibLINEAR, LibSVM, LibSVMLoader, LibSVMSaver, LinearForwardSelection, LinearNNSearch, LinearRegression, LinearRegression, LinearUnit, ListOptions, Literal, LiteralSet, LMT, LMTNode, LocalScoreSearchAlgorithm, Logger, Logistic, LogisticBase, LogitBoost, LovinsStemmer, LUDecomposition, LWL, M5Base, M5P, M5Rules, MahalanobisEstimator, MakeDecList, MakeDensityBasedClusterer, MakeIndicator, ManhattanDataObject, ManhattanDistance, MarginCalculator, MarginCalculator.JunctionTreeNode, MarginCalculator.JunctionTreeSeparator, MarginCurve, MathematicalExpression, MathExpression, Maths, Matrix, Matrix, MDD, MedianDistanceFromArbitraryPoint, MedianOfWidestDimension, MedianOfWidestDimension, Memory, MergeTwoValues, MetaCost, MethodHandler, MexicanHat, MIBoost, MIDD, MiddleOutConstructor, MidPointOfWidestDimension, MIEMDD, MILR, MINND, MIOptimalBall, MIPolyKernel, MIRBFKernel, MISMO, MISVM, MIWrapper, MixtureDistribution, ModelSelection, MultiBoostAB, MultiClassClassifier, MultiFilter, MultiInstanceToPropositional, MultilayerPerceptron, MultiNomialBMAEstimator, MultipleClassifiersCombiner, MultiScheme, NaiveBayes, NaiveBayes, NaiveBayesMultinomial, NaiveBayesMultinomialUpdateable, NaiveBayesSimple, NaiveBayesUpdateable, NBTree, NBTreeClassifierTree, NBTreeModelSelection, NBTreeNoSplit, NBTreeSplit, ND, NDConditionalEstimator, NearestNeighbourSearch, NeuralConnection, NeuralNetwork, NeuralNode, NGramTokenizer, NNConditionalEstimator, NNge, NominalPrediction, NominalToBinary, NominalToBinary, NominalToString, NonSparseToSparse, NormalEstimator, NormalizableDistance, Normalize, Normalize, NormalizedPolyKernel, NormalMixture, NoSplit, NullStemmer, NumericCleaner, NumericPrediction, NumericToBinary, NumericToNominal, NumericTransform, Obfuscate, OneR, OneRAttributeEval, OPTICS, OPTICS\_Visualizer, Optimization, Option, OptionHandlerJavadoc, OrdinalClassClassifier, OutputLogger, OutputZipper, PaceMatrix, PaceRegression, PairedCorrectedTTester, PairedStats, PairedStatsCorrected, PairedTTester, ParentSet, PART, PartitionedMultiFilter, PerformanceStats, PKIDiscretize, PLSClassifier, PLSFilter, PMMLClassifier, PointsClosestToFurthestChildren, PoissonEstimator, PolyKernel, PotentialClassIgnorer, PrecomputedKernelMatrixKernel, PreConstructedLinearModel, Predicate, PredictionNode, PredictiveApriori, PrincipalComponents, PrincipalComponents, Prior, PriorEstimation, PriorityQueue, PriorityQueueElement, Prism, PropertyHandler, PropertyNode, PropertyPath, PropertyPath.Path, PropertyPath.PathElement, PropositionalToMultiInstance, ProtectedProperties, PruneableClassifierTree, PruneableDecList, Puk, QRDecomposition, Queue, RacedIncrementalLogitBoost, RaceSearch, RandomCommittee, RandomForest, RandomizableClassifier, RandomizableClusterer, RandomizableDensityBasedClusterer, RandomizableIteratedSingleClassifierEnhancer, RandomizableMultipleClassifiersCombiner, RandomizableSingleClassifierEnhancer, RandomizableSingleClustererEnhancer, Randomize, RandomProjection, RandomRBF, RandomSearch, RandomSplitResultProducer, RandomSubset, RandomSubSpace, RandomTree, RandomVariates, Range, Ranker, RankSearch, RBFKernel, RBFNetwork, RDG1, ReferenceInstances, RegOptimizer, Regression, RegressionByDiscretization, RegressionGenerator, RegressionSplitEvaluator, RegSMO, RegSMOImproved, RELAGGS, RelationalLocator, ReliefFAttributeEval, RemoteEngine, RemoteExperiment, RemoteExperimentSubTask, Remove, RemoveFolds, RemoveFrequentValues, RemoveMisclassified, RemovePercentage, RemoveRange, RemoveType, RemoveUseless, RemoveWithValues, Reorder, RepeatedHillClimber, RepeatedHillClimber, ReplaceMissingValues, REPTree, Resample, Resample, ReservoirSample, ResidualModelSelection, ResidualSplit, ResultMatrix, ResultMatrixCSV, ResultMatrixGnuPlot, ResultMatrixHTML, ResultMatrixLatex, ResultMatrixPlainText, ResultMatrixSignificance, ResultVectorTableModel, Ridor, RotationForest, Rule, Rule, Rule, RuleGeneration, RuleItem, RuleNode, RuleStats, ScatterSearchV1, SearchAlgorithm, SelectedTag, Sequence, SequentialDatabase, SERFileFilter, SerializationHelper, SerializedClassifier, SerializedInstancesLoader, SerializedInstancesSaver, SerializedObject, SerialUIDChanger, SERObject, sIB, SigmoidUnit, SimpleBatchFilter, SimpleCart, SimpleEstimator, SimpleFilter, SimpleKMeans, SimpleLinearRegression, SimpleLinkedList, SimpleLinkedList.LinkedListInverseIterator, SimpleLinkedList.LinkedListIterator, SimpleLogistic, SimpleMI, SimpleStreamFilter, SimulatedAnnealing, SimulatedAnnealing, SingleAssociatorEnhancer, SingleClassifierEnhancer, SingleClustererEnhancer, SingleIndex, SingularValueDecomposition, SlidingMidPointOfWidestSide, SMO, SMOreg, SMOset, SMOTE, SnowballStemmer, SparseInstance, SparseToNonSparse, SpecialFunctions, SPegasos, SplitCriterion, Splitter, SpreadSubsample, Stack, Stacking, StackingC, Standardize, Statistics, Stats, Stats, Stemming, Stopwords, StratifiedRemoveFolds, StringKernel, StringLocator, StringToNominal, StringToWordVector, SubsetByExpression, SubsetSizeForwardSelection, SubspaceCluster, SubspaceClusterDefinition, SVMAttributeEval, SVMLightLoader, SVMLightSaver, SwapValues, SymmetricalUncertAttributeEval, SystemInfo, TabuSearch, TabuSearch, Tag, TAN, TAN, TaskStatusInfo, TechnicalInformation, TechnicalInformationHandlerJavadoc, Tee, Tertius, Test, TestInstances, TextDirectoryLoader, ThresholdCurve, ThresholdSelector, TimeSeriesDelta, TimeSeriesTranslate, Tokenizer, TopDownConstructor, TreePerformanceStats, Trie, Trie.TrieIterator, Trie.TrieNode, TwoClassStats, TwoWayNominalSplit, TwoWayNumericSplit, UnsupervisedAttributeEvaluator, UnsupervisedSubsetEvaluator, UpdateQueue, UpdateQueueElement, UserClassifier, Utils, Values, VaryNode, Version, VFI, Vote, VotedPerceptron, WAODE, Wavelet, Winnow, WordTokenizer, WrapperSubsetEval, XMeans, XMLBasicSerialization, XMLBeans, XMLClassifier, XMLDocument, XMLExperiment, XMLInstances, XMLOptions, XMLSerialization, XMLSerializationMethodHandler, XRFFLoader, XRFFSaver, XStream, YongSplitInfo, ZeroR

---

``` public interface RevisionHandler ```

For classes that should return their source control revision.

**Version:**
:   $Revision: 1.1 $

**Author:**
:   fracpete (fracpete at waikato dot ac dot nz)

**See Also:**: `RevisionUtils`

---

| **Method Summary** | |
| --- | --- |
| `java.lang.String` | `getRevision()`             Returns the revision string. |

**Method Detail**

### getRevision

```
java.lang.String getRevision()
```

:   Returns the revision string.

    :   **Returns:**: the revision


---


|  |  |  |
| --- | --- | --- |
| **Overview** \| **Package** \| **Class** \| **Tree** \| **Deprecated** \| **Index** \| **Help** \| **Weka's home** | |  |
| **PREV CLASS**   **NEXT CLASS** | **FRAMES**    **NO FRAMES**     **All Classes** |  |
| SUMMARY: NESTED \| FIELD \| CONSTR \| METHOD | DETAIL: FIELD \| CONSTR \| METHOD |  |


---
